# Discovery of 3CLpro inhibitor of SARS-CoV-2 main protease

**DOI:** 10.2144/fsoa-2023-0020

**Published:** 2023-04-06

**Authors:** Yi Kuang, Xiaodong Ma, Wenjing Shen, Qingqing Rao, Shengxiang Yang

**Affiliations:** 1 College of Chemical & Materials Engineering, Zhejiang A&F University, Lin'an, Zhejiang, 311300, PR China

**Keywords:** 3CLpro, ADMET prediction, molecular docking, molecular dynamics simulation, SARS-CoV-2

## Abstract

Coronavirus main protease (3CLpro), a special cysteine protease in coronavirus family, is highly desirable in the life cycle of coronavirus. Here, molecular docking, ADMET pharmacokinetic profiles and molecular dynamics (MD) simulation were performed to develop specific 3CLpro inhibitor. The results showed that the 137 compounds originated from Chinese herbal have good binding affinity to 3CLpro. Among these, Cleomiscosin C, (+)-Norchelidonine, Protopine, Turkiyenine, Isochelidonine and Mallotucin A possessed prominent drug-likeness properties. Cleomiscosin C and Turkiyenine exhibited excellent pharmacokinetic profiles. Furthermore, the complex of Cleomiscosin C with SARS-CoV-2 main protease presented high stability. The findings in this work indicated that Cleomiscosin C is highly promising as a potential 3CLpro inhibitor, thus facilitating the development of effective drugs for COVID-19.

## Plain language summary

In this work, computer aided drug design technology was used to study the main protease 3CLpro of novel coronavirus, and functional small molecules with inhibitory effects on novel coronavirus were screened from the compound library of natural products. The results showed that Cleomiscosin C is highly promising as a potential 3CLpro inhibitor with prominent binding affinity, pharmacokinetic profiles and stability.

As a new type of positive strand RNA virus β-Coronavirus [1], SARS-CoV-2 is still characterized by high mutation rate. People infected with this virus will have fever, dry cough, fatigue, shortness of breath, and other symptoms [2]. According to “Our World in Data” from Oxford University [3], a total of 12,123,516,491 doses of novel coronavirus vaccine have been administered worldwide so far, with a vaccination rate of 66.72%. Nevertheless, many virus variants have occurred due to the high mutation characteristics of coronavirus [4], thus greatly reducing the effect of antiviral drugs and vaccines. Therefore, developing novel antiviral drugs and vaccines is extremely significant.

At present, oral anti COVID-19 drugs are mainly divided into 3CLpro inhibitors and RNA-dependent-polymerase (RdRp) inhibitors, which are developed for different key targets of the virus life cycle. For example, Paxlovid developed by Pfizer is a 3CLpro inhibitor. Remdesivir, the first officially approved COVID-19 therapeutic drug by FDA and Molnupiravir are both the RdRp inhibitors. In addition, Dasabuvir was found to be a potential molecule for both PLpro and 3CLpro throughout molecular docking, MD simulation and MM/PBSA analysis within 53 FDA approved antiviral drugs [5].

**Figure 1.**
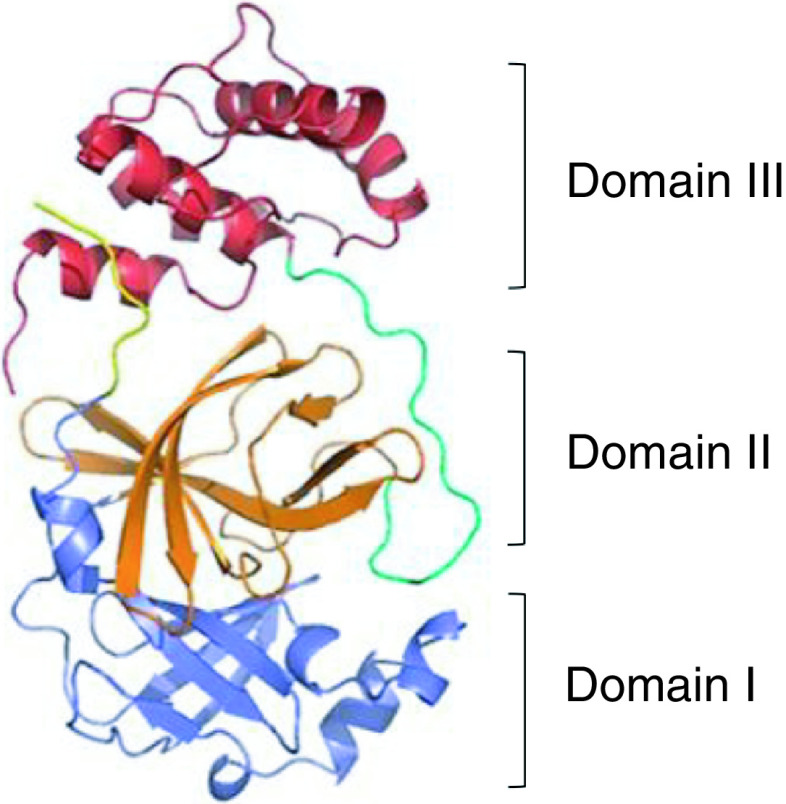
The structure of SARS-CoV-2 3Clpro. The N-finger (residues 1-7) is depicted in yellow, domain I (residues 8–101) in blue, domain II (residues 102–184) in orange, the loop region (residues 185–200) in green, domain III (residues 201-306) in red.

3-Chymotrypsin like protease (3CLpro) [6,7] is a homodimeric cysteine protease consisting of 306 amino acids and 3 domains, which can cleave coronavirus polyproteins at 11 conservative sites [8]. As shown in Figure 1, antiparallel β-barrel is the main secondary structure of domains I (residues 8–101) and II (residues 102-184) [9,10]. Domain III is an additional helical domain, its aggregation triggered the dimerization of 3CLpro, resulting in a mature dimer structure. Domain III is connected to domain II by long linker group (residues 185–200). The first seven residues from the N-terminus side are forming the N-finger, which is believed to have a significant role both in dimerization and in establishing proteolytic activity [11]. The viral RNA was initially translated into polyproteins, and then cut into multiple active functional proteins by 3CLpro when it entered into human cells, such as viral replication protein RdRp, which participated in the replication of viral RNA. However, without the hydrolysis and cleavage of 3CLpro, the subsequent translation and replication cannot be carried out, and the generation of offspring viruses cannot continue even if the SARS-CoV-2 virus entered into the cells. As a result, inhibiting the activity of 3CLpro protease is capable of preventing virus replication. In addition, there is no reporting about the human homologues of 3CLpro, suggesting the negligible inhibition of 3CLpro inhibitors on human protease. Consequently, 3CLpro is an ideal anti-virus target.

Chinese herbal medicine is an important part of traditional Chinese medicine. Recently, numerous researches have shown that a variety of natural compounds in Chinese herbal medicines have excellent biological activities favorable efficacy in anti-tumor, anti-inflammatory and antibacterial [12]. In addition, the natural compounds in Chinese herbal medicine have a certain effect on the treatment and control of COVID-19 [13]. Up to now, Chinese herbal medicine has made many great achievements in the medicine industry. For example, artemisinin developed by Tu Youyou, a Nobel laureate in medicine, has significantly reduced the death rate of malaria patients [14]. In addition, paclitaxel has been proved to have excellent anti-tumor, anti-diabetes and anti-fungal effects [15], also, the cephaletin has been found as a potential COVID-19 drug [16]. The National Collection of Chinese Herbal Medicine contains a total of more than 4000 kinds of Chinese herbal medicine. More than 500 kinds of toxic traditional Chinese medicines were screened to make a list of toxic traditional Chinese medicines.

Virtual screening [17] based on molecular docking, pharmacokinetic and molecular dynamics refers to simulating the interaction between targets before biological activity screening, analyzing the possible related reactions between drugs and the body, so as to improve the discovery efficiency of lead compounds and improve the success rate of drug research and development. In 2020, through homology modeling, molecular docking and binding free energy calculation, Nelfinavir was found to have therapeutic effects on COVID-19.

Herein, 1047 natural compounds were collected from 34 Chinese herbal medicines as ligands to screen on the potential 3CLpro inhibitors. Firstly, molecular docking technology was performed to evaluate the binding free energy of natural compounds-3CLpro complex, screening out the compounds with prominent binding affinity with 3CLpro. Then the drug-likeness properties and pharmacokinetic characteristics of the selected compounds were further evaluated. Afterward, molecular dynamics simulation was employed to estimate the complex stability. Finally, the most promising 3CLpro inhibitors were confirmed according to the comprehensive performance evaluation.

## Materials & methods

### Molecular docking

Previous research has identified that the compound ML188 was an inhibitor of the SARS-CoV 3CLpro [18]. As a non-covalent inhibitor, ML188 [19] was found to be more effective in inhibiting SARS-CoV-2 3CLpro at 2.5 μM than that of inhibiting SARS-CoV 3CLpro. Therefore, the protein 7L0D [20] containing the co-crystalline ligand ML188 was selected as the target.

The x-ray crystal three-dimensional structure of SARS-CoV-2 main protease 3CLpro (PDB ID: 7L0D, resolution: 2.39 Å) in complex with a non-covalent inhibitor ML188 was taken from the Protein Data Bank [21]. Solvents, water molecules, other heteroatoms and ligand in protein were removed by using Pymol software. AutoDock Tools was used to add polar hydrogens to the structures, and then converting to pdbqt format. Co-crystallized ligand of 7L0D (ML188), as a control in docking studies, was extracted by using Pymol software, and then converted to corresponding formats.

The ligands used for molecular docking were selected from the catalogue of toxic Chinese herbal medicines in the national compilation of Chinese herbal medicines [22]. The corresponding natural compound structure files were downloaded from PubChem database according to the CAS number provided in the literature. Ligands were converted to PDB format using Open Babel software [23] and further prepared to pdbqt format using AutoDock Tools.

Docking simulations were performed with AutoDock Vina [24] using the empirical free energy force field and Lamarckian genetic algorithm conformational search with default parameters [25]. The ideal active site where the non-covalent ML188 inhibitor interacts with 3CLpro in the 7L0D complex was chosen as the lead-in site to screen the interaction and affinity of herbal ligands with 3CLpro, taking the similar strategy that has been described previously in several studies [26–30]. The grid coordinates of protein ligand binding sites are as follows, x = 11.701 Å, y = -17.509 Å, z = 16.43 Å, while grid dimensions were set at 60 × 40 × 60 Å3, with a spacing of 0.775 Å between the grid points. Other parameters were set to default [31,32]. The interactions of protein–ligand predicted by docking researches were visualized by Pymol. The 2D-schematic diagram of protein–ligand interaction was generated using LigPlot [33].

### *In silico* prediction of molecular properties & pharmacokinetic ADMET profiles

A large number of potential therapeutic agents fail to reach clinical trials due to adverse parameters of ADMET [34,35]. Thus, testing these properties is useful for drug discovery. The molecular properties [36] and ADMET properties [37,38] of the promising natural compounds were estimated using the online web server pkCSM [39] and SwissADME [40].

MW (Molecular weight), TPSA (topological polar surface area), Log-P (lipophilicity), N.HBA (number of hydrogen bond acceptors), N.HBD (number of hydrogen bond donors), N.RB (number of rotational bonds) were analyzed to evaluate the drug likeness of the potential inhibitors. If Log-p > 5, this deviation results in low solubility and poor oral absorption. For TPSA, when it is less than 140 Å and the number of rotatable bonds is less than 10, the compounds become more flexible and is more able to interact with the target receptor.

ADMET properties such as Water solubility, Intestinal absorption (human), VDss (human), Caco-2 permeability [41], BBB permeability [42,43], CYP3A4 inhibitor [44], CNS permeability and Hepatotoxicity [45] were calculated for drug screening and development. Intestine is the primary site for an orally administered drug absorption, a molecule with an absorbance of less than 30% is considered to be poorly absorbed. In terms of distribution indicators, the distribution (VDss) size is considered high if its value is greater than 0.45, the higher the VDss is, the more of a drug is distributed in tissue rather than plasma. For the CNS index, compounds with LogPS < -3 are believed to be incapable of penetrating the CNS.

### Molecular dynamics (MD) simulations

Molecular dynamics simulation [46,47] of protein–ligand complexes was performed using Gromacs software [48], the protein–ligand complexes were prepared by using Charmm36 [49] forcefield, and the ligand topology was generated by Swissparam [50].

First, download the sdf file of the ligand from Pubchem according to the CAS number, then open the file with Avogadro program, hydrogenate it, and save it as mol2 file. Finally, the file is submitted to Swissparam for processing.

A solvated dodecahedron box of SPC [51] water model with 1 nm distances was constructed between the box edge and atoms of the protein–ligand complexes. And 4 sodium (Na+) were inserted to neutralize all solvated systems. Afterward, the systems were minimized through 50000 steps of the steepest descent algorithm [52]. Furthermore, 100 ps NVT balance and 100 ps NPT balance were carried out to make the system reach a stable state 300 K and 1 bar [53]. Finally, 100 ns molecular dynamics simulations were carried out toward the complex.

Then the main parameters, such as root mean square deviation (RMSD) [54,55], root mean square fluctuation (RMSF) [56], radius of gyration (Rg) [57] and number of hydrogen bonds [58] were performed to evaluate the stability of protein–ligand complexes [59].

## Results

### Protein contact atlas analysis

**Figure 2.**
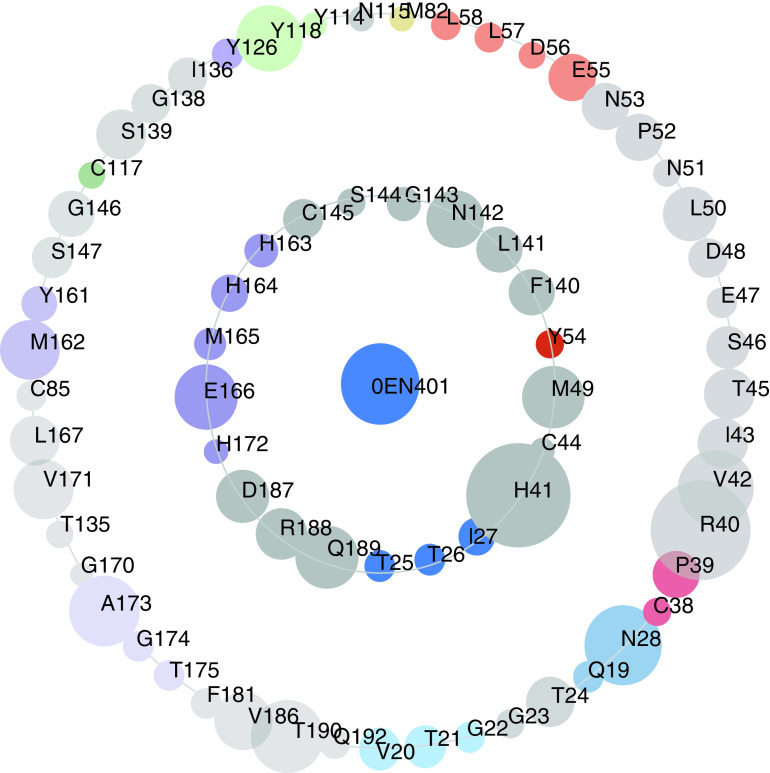
Visualization and analysis of protein–ligand contacts. The asteroid plots of ML188 co-crystal structure (PDB ID: 7L0D). Asteroid plot with the ligand highlighted in blue (central node). Directly contacting residues (first-shell residues) are shown in the inner circle, and the residues that contact these but not the ligand (second-shell residues) are shown in the outer circle. The residues are colored according to their secondary structures, and the size of each circle is scaled to denote the number of atomic contacts.

The asteroid plot is an innovative method for analyzing the interactions between protein and ligand based on the multilevel visualization of noncovalent contacts [60]. Here, the representative co-crystal structure 7L0D of 3CLpro and the key amino acids of this structure were obtained from the online website Protein-Contact-Atlas (Figure 2).

### Molecular docking of 3CLpro inhibitors

**Figure 3.**
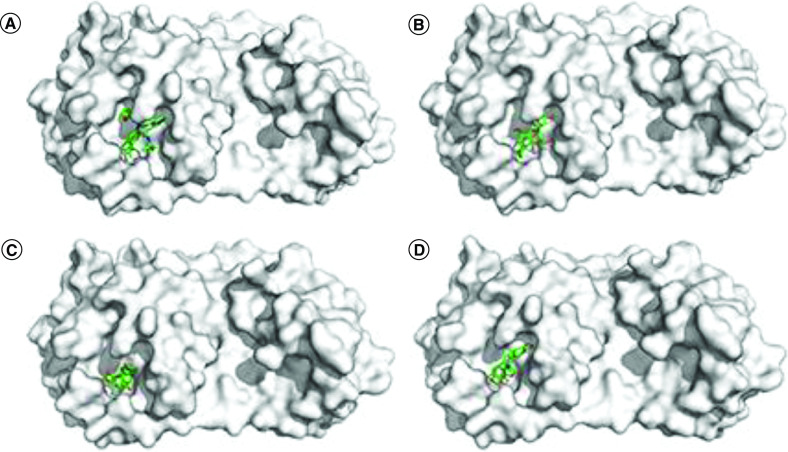
The docking poses of compounds (A) ML188, (B) Cleomiscosin C, (C) (+)-Norchelidonine and (D) Turkiyenine and within SARS-CoV-2 3CLpro (7LOD) and hydrophobicity surface of 3CLpro active site with these ligands.

Table 1 listed the docking scores of partially natural compounds. As a positive control, the docking scores of co-crystal ligand ML188 was -8.1 kcal/mol. The specific docking modes [61] of three natural compounds, Cleomiscosin C, (+)-Norchelidonine and Turkiyenine were exhibited in Figure 3.

**Table 1.** Docking fraction of part compounds.

Compound	Docking Score (kcal/mol)
Helioscopinin A	-11.1
Chelidimerine	-10.5
Plumbazeylanone	-10.3
Kayaflavone	-9.9
Furosin	-9.6
Cyclotrijuglone	-9.5
Isoquercetrin	-9.3
**Turkiyenine**	-9.1
Tellimagrandin I	-9.1
Indioside A	-9
Euphorbin D	-9
**(+)-Norchelidonine**	-8.7
Solamargine	-8.7
Quercetin-3-Arabinoside	-8.7
**Protopine**	-8.6
**Cleomiscosin C**	-8.6
Heveaflavone	-8.4
**Mallotucin A**	-8.2
**Isochelidonine**	-8.2
ML188	-8.1

Compounds in bold were selected for subsequent pharmacokinetic and molecular dynamics simulations.

**Figure 4.**
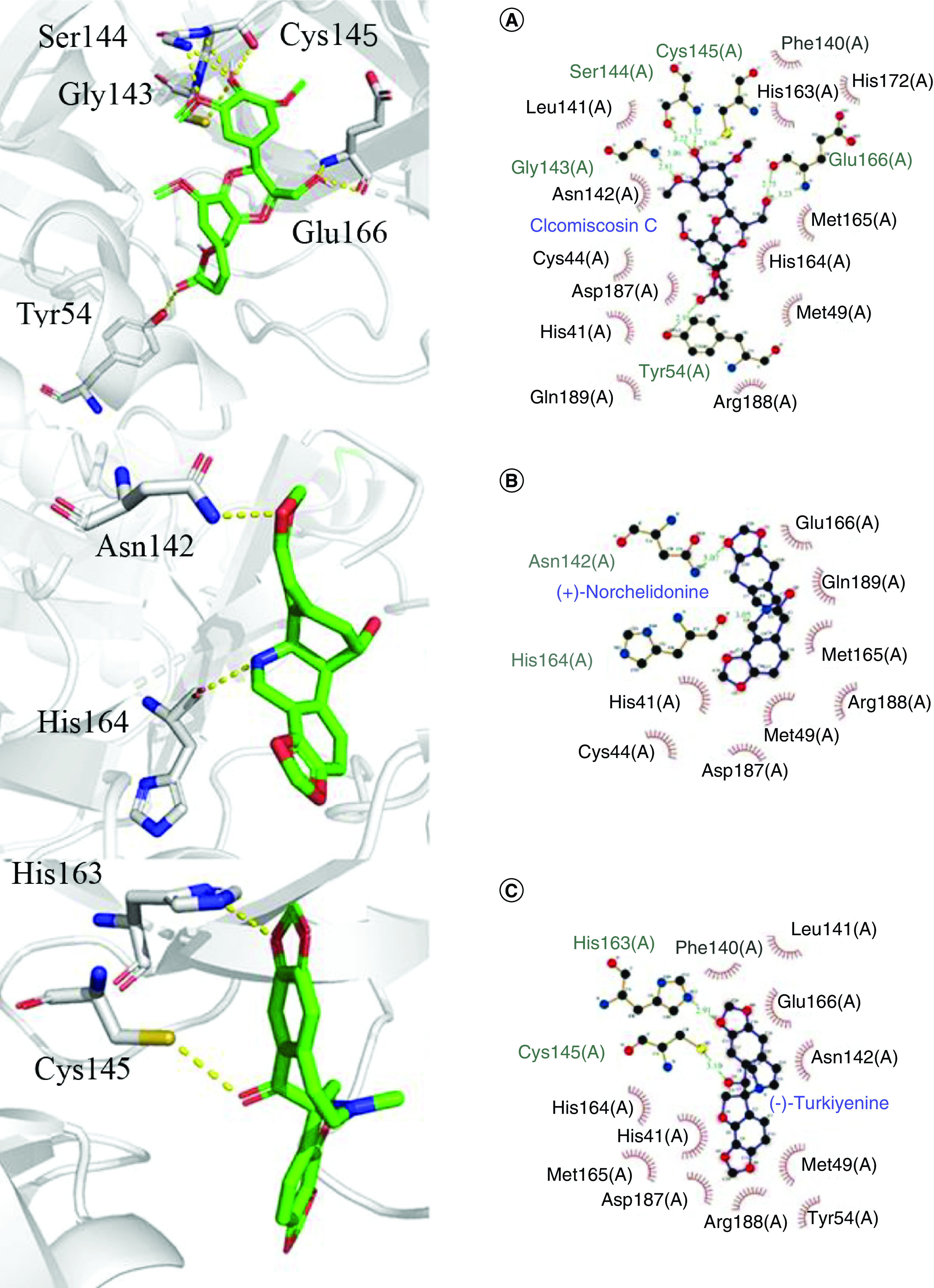
3D and 2D molecular interactions of the predicted docking poses of compounds (A) Cleomiscosin C, (B) (+)-Norchelidonine, and (C) Turkiyenine toward SARS-CoV-2 3CLpro.

For Cleomiscosin C [62], a compound from Xanthium sibiricum, displayed eight hydrogen bonds with the key amino acid residues of 3CLpro at Tyr54, Gly143, Ser144, Cys145 and Glu166, respectively, and the docking score was -8.6 kcal/mol. In addition, hydrophobic interactions were found between the amino acid residues and Cleomiscosin C at His41, Cys44, Met49, Phe140, Leu141, Asn142, His163, His164, Met165, His172, Asp187, Arg188 and Gln189 (Figure 4).

For (+)-Norchelidonine [63], a compound from Chelidonium majus, only showed two hydrogen bonds with the key amino acid residues of 3CLpro, Asn142 and His164 with bond lengths of 3.07 Å and 3.05 Å, respectively, and the docking score was -8.7 kcal/mol. Similarly, hydrophobic interactions were found between the amino acid residues and (+)-Norchelidonine at His41, Cys44, Met49, Met165, Glu166, Asp187, Arg188 and Gln189 (Figure 4).

For Turkiyenine [64], also a compound from Chelidonium majus, showed two hydrogen bonds with the key amino acid residues of 3CLpro at Cys145 and His163, respectively, and the docking score was -9.1 kcal/mol. As presented in Figure 4, there was also a hydrophobic interaction between the amino acid residues and Turkiyenine at His41, Met49, Tyr54, Phe140, Leu141, Asn142, Chis164, Met165, Glu166, Asp187 and Arg188.

### *In silico* prediction of molecular properties & pharmacokinetic ADMET profiles

Further these 137 natural compounds selected with molecular docking results were evaluated by Lipinski's rules [65]. Table 2 listed the molecular properties of Cleomiscosin C, (+)-Norchelidonine, Protopine, Turkiyenine, Isochelidonine and Mallotucin A.

**Table 2.** Portended physiochemical parameters of the potential inhibitors.

Compound	MW	TPSA	Log-P	N.RB	N.HBA	N.HBD
Cleomiscosin C	416.382	116.82	2.3978	5	9	2
(+)-Norchelidonine	339.347	69.18	1.9891	0	6	2
Protopine	353.374	57.23	2.5573	0	6	0
Turkiyenine	365.341	66.46	2.5307	0	7	0
Isochelidonine	353.374	60.39	2.3313	0	6	1
Mallotucin A	328.364	65.74	3.3159	1	5	0

MW: Molecular weight; N.HBA: Number of hydrogen bond acceptors; N.HBD: Number of hydrogen bond donors; N.RB: Number of rotational bonds; TPSA: Topological polar surface area.

Thereafter, *in silico* ADMET analysis was employed to predict the pharmacokinetic profile of compounds. As stated in the Table 3, Cleomiscosin C has a 100% absorption value and is better than the other 5 compounds in human intestinal absorption. Cleomiscosin C was an inhibitor of P-glycoprotein (P-gp I and P-gp II), but the other five compounds did not meet this index, and Mallotocin A was neither a P-gp I inhibitor nor a P-gp II inhibitor. Besides, since the inability of Cleomiscosin C and (+)-Norchelidonine to permeate across the blood–brain barrier (BBB), the central nervous system can be effectively protected from the effects of drugs. Only CNS permeability of Cleomiscosin C is less than -3, indicating that it cannot penetrate the central nervous system and can better protect the human body. Notably, cytochrome P450 is an important detoxification enzyme in the body, mainly found in the liver, this enzyme oxidizes foreign microorganisms to facilitate their excretion. The results displayed that Cleomiscosin C and Mallotucin A did not perform as inhibitors for CYP1A2, CYP2C19, CYP2C9, CYP2D6 and CYP3A4 enzymes, but (+)-Norchelidonine and Protopine were the inhibitors of two CYPs, Turkiyenine and Isochelidone were one of the inhibitors. Moreover, the Maximum Tolerated Dose and Hepatotoxicity tests of Cleomiscosin C, (+)-Norchelidonine, Protopine, Turkiyenine showed no adverse effects.

**Table 3.** Anticipated ADMET characteristics of the potential inhibitors.

ADME Parameters	Cleomiscosin C	(+)-Norchelidonine	Turkiyenine	Protopine	Isochelidonine	Mallotucin A
Water solubility	-3.462	-3.047	-3.782	-3.825	-3.984	-4.286
Caco-2 permeability	0.31	1.065	1.336	1.065	0.934	0.999
Intestinal absorption (human)	100	93.194	95.282	97.593	94.711	99.164
P-glycoprotein I inhibitor	Yes	No	Yes	Yes	Yes	No
P-glycoprotein II inhibitor	Yes	Yes	No	No	No	No
VD_ss_ (human)	0.109	0.986	-0.206	0.696	0.682	0.173
BBB permeability	No	No	Yes	Yes	Yes	Yes
CNS permeability	-3.613	-2.273	-2.859	-1.977	-2.108	-2.912
CYP1A2 inhibitor	No	Yes	Yes	Yes	Yes	No
CYP2C19 inhibitor	No	Yes	No	Yes	No	No
CYP2C9 inhibitor	No	No	No	No	No	No
CYP2D6 inhibitor	No	No	No	No	No	No
CYP3A4 inhibitor	No	No	No	No	No	No
AMES toxicity	No	No	Yes	Yes	No	No
Max. tolerated dose (human)	-0.187	-0.458	-0. 079	-0.446	0.028	-0.158
Hepatotoxicity	No	No	No	No	Yes	Yes
Skin sensitisation	No	No	No	No	No	No
Minnow toxicity	1.92	1.499	2.051	1.237	1.553	0.886

### Molecular dynamics simulations

Subsequently, molecular dynamics simulations, a calculation method for imitating the physical trajectory and state of atoms and molecules based on Newtonian mechanics, were performed to analyze the dynamic behavior of complex systems. The structural stability of Cleomiscosin C, (+)-Norchelidonine and Turkiyenine in complex with 3CLpro were simulated by molecular dynamics in this work, employing the co-crystal inhibitor (ML188) as the control group.

#### Root mean square deviation

**Figure 5.**
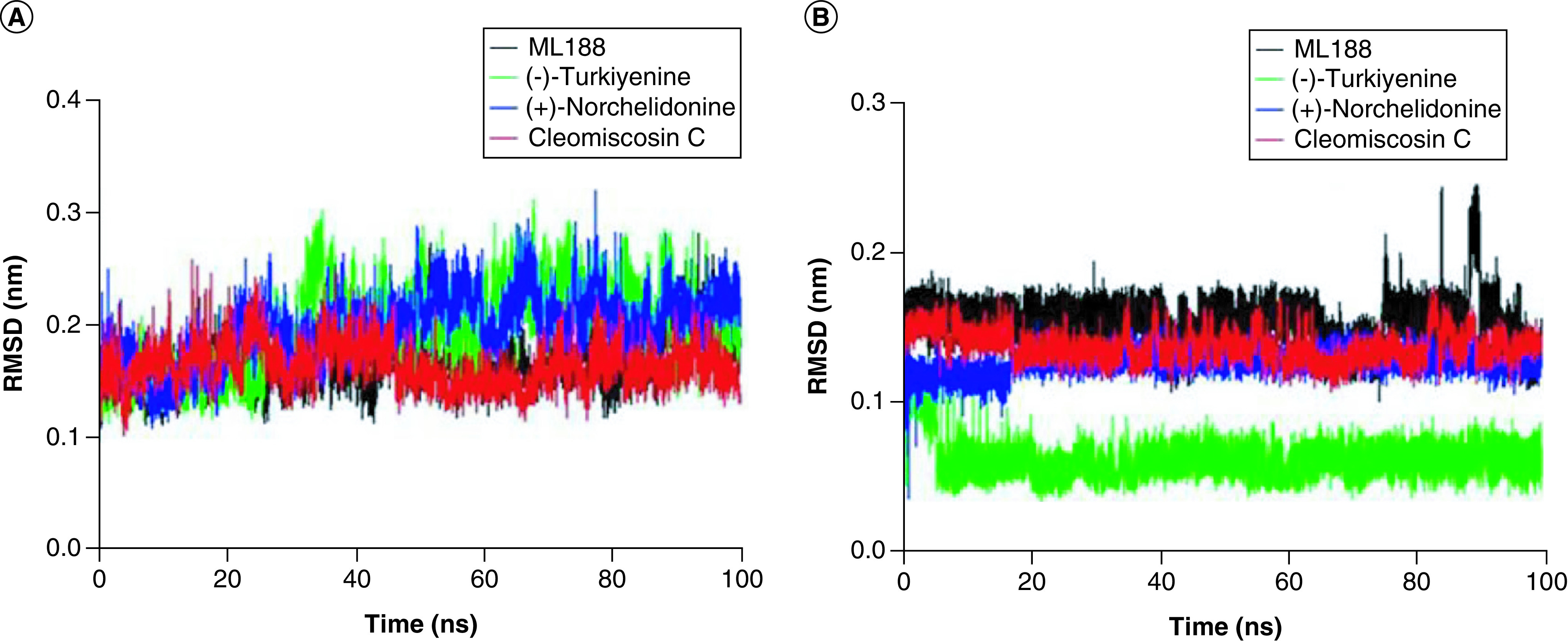
The root mean square deviation profiles of protein backbones (A) and free ligands Cleomiscosin C, (+)-Norchelidonine, Turkiyenine and control ML188 (B) for SARS-CoV-2 3CLpro complexes. RMSD: Root mean square deviation.

As shown in Figure 5A, the RMSD value of 3CLpro complex with ligand Cleomiscosin C fluctuated from 0.1 to 0.2 nm, with an average of 0.16 nm, has also showing a similar trend with ML188 complex. In addition, the mean RMSD values for 3CLpro in complex with (+)-Norchelidonine and Turkiyenine were 0.19 nm and 0.20 nm, respectively, with a slightly larger range of fluctuations.

In addition, the RMSD values of the ligand Turkiyenine was lower than 0.1 nm, and the RMSD values of the ligands Cleomiscosin C and (+)-Norchelidonine showed no obvious change throughout the simulation process and fluctuated around 0.15 nm, suggesting the good binding ability of ligands with the active site on 3CLpro (Figure 5B).

#### Root mean square fluctuation

**Figure 6.**
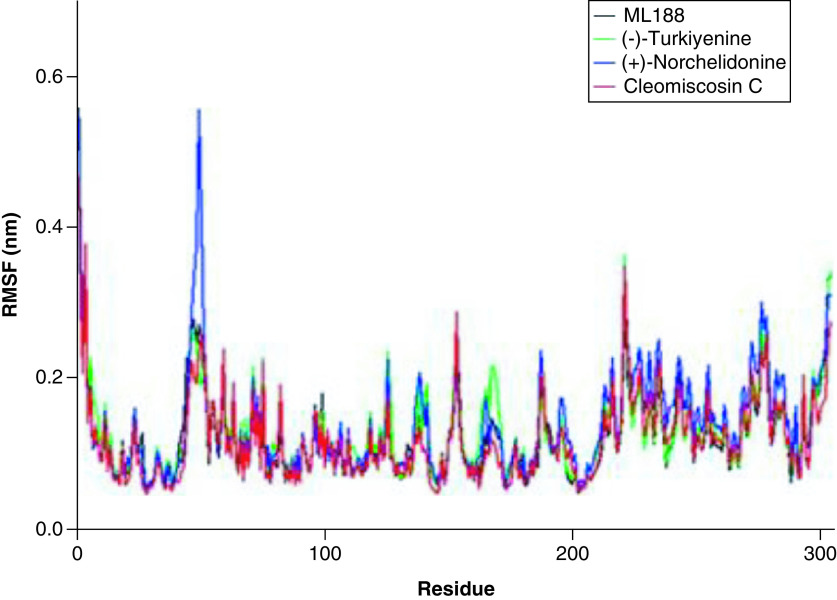
The RMSF profiles of SARS-CoV-2 3CLpro complexes with potential inhibitors Cleomiscosin C, (+)-Norchelidonine and Turkiyenine. RMSF: Root mean square fluctuation.

Moreover, the RMSF value of (+)-Norchelidonine complex with 3CLpro showed significant fluctuations at ASP-48, MET-49, LEU-50 and ASN-51. Similarly, Turkiyenine complex with 3CLpro was also unstable during the simulation process, the obvious fluctuations of RMSF value of Turkiyenine-3CLpro complex were found at ARG-222, PHE-223, and THR-224. However, Cleomiscosin C-3CLpro complex exhibited superb stability and the RMSF value of key amino acid residues is small (Figure 6). Notably, there is no obvious structural change during the whole simulation process, indicating the excellent effect of Cleomiscosin C-3CLpro complex.

#### Radius of gyration

**Figure 7.**
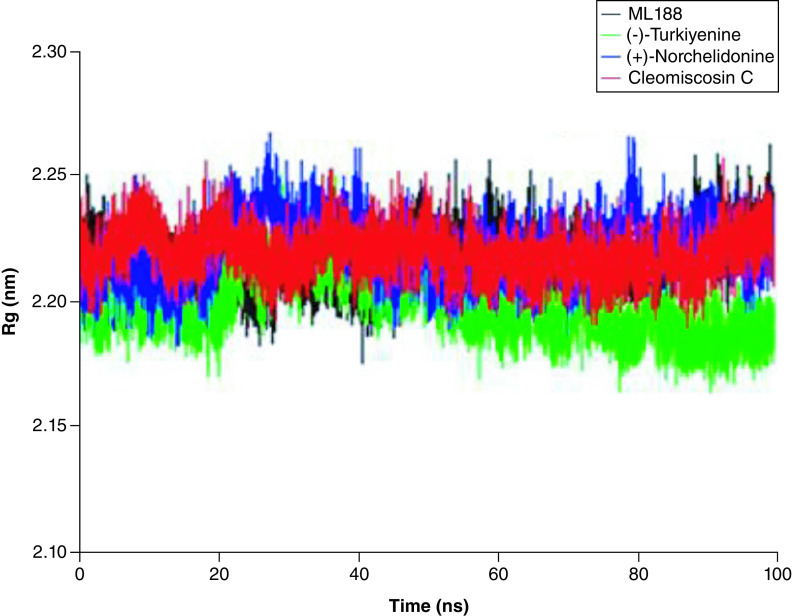
Backbone radius of gyration of 3CLpro as free proteins and forming complexes with ligands Cleomiscosin C, (+)-Norchelidonine and Turkiyenine. Rg: Radius of gyration.

Radius of gyration is an important index to characterize the compactness of protein structure and the change of protein looseness during simulation procedure. The abrupt fluctuation of RG values indicates the instability of protein structural. As shown in Figure 7, these three complexes presented prominent structural stability during the whole simulation process, the RG value of them almost remained unchanged within 100 ns, and the fluctuation range was only about 0.05 nm.

#### Hydrogen bonds

**Figure 8.**
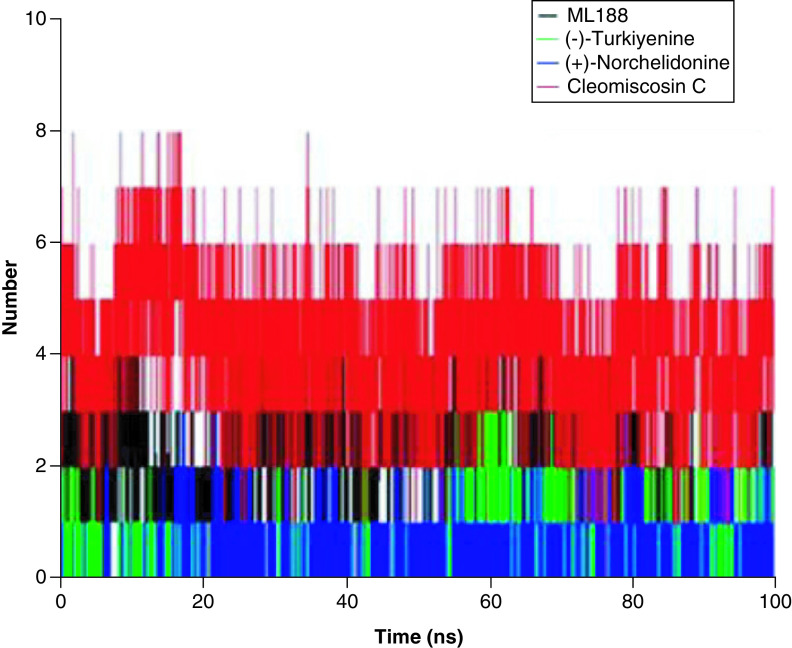
Number of hydrogen bonds formed between Cleomiscosin C, (+)-Norchelidonine and Turkiyenine and SARS-CoV-2 3CLpro.

Hydrogen bond is an important part of intermolecular force of protein–ligand complexes. The more hydrogen bonds formed in protein–ligand complexes, the stronger interaction between them. It should be pointed out that different software has different determination mechanisms for hydrogen bonds. The determination of hydrogen bonds in GROMACS software needs to meet two standards: (1) hydrogen bond distance r ≤ 0.35 nm; (2) angle alpha ≤30°. As shown in Figure 8, the number of hydrogen bonds fluctuated throughout the 100 ns MD simulations, and the average number of hydrogen bonds was 2, 4, 1 and 1 for ML188-, Cleomiscosin C-, (+)-Norchelidonine-, and Turkiyenine-3CLpro complexes, respectively. This result was highly consistent with the number of hydrogen bonds determined by Ligplot, suggesting the high affinity and stability of Cleomiscosin C-3CLpro complex.

## Discussion

In searching for small compounds to prohibit viral replication and transcription, virtual screening techniques were utilized to explore a chemical library from the toxic Chinese herbal medicines as potential SARS-CoV-2 3CLpro inhibitors.

The co-crystal ligand ML188 forms strong interactions with the direct contacted residues, such as: His41, Met49, Asn142, Glu166, Gln189, Arg188 and Asp187 in providing vital information for the following evaluation of the interaction patterns of the screened compounds.

Molecular docking results showed that, 137 natural compounds with docking scores less than -8.1 kcal/mol. Among them, Cleomiscosin C, (+)-Norchelidonine and Turkiyenine exhibited strong hydrogen bonds and hydrophobic interactions.

The drug-likeness is the most recent method proposed to identify compounds that are recommended for use in drugs that must respect Lipinski's rules, compounds need to conform to the following properties: molecular weight <500; N.HBD <5; N.HBA <10; N.RB <10; and the logarithm of octanol-water partition coefficient (Log-P) between -2 to 5. Among the 137 natural compounds with docking scores less than -8.1 kcal/mol, only Cleomiscosin C, (+)-Norchelidonine, Protopine, Turkiyenine, Isochelidonine and Mallotucin A complyed with Lipinski's rules. *In silico* ADMET analysis, Cleomiscosin C had the best pharmacokinetic profiles, followed by Turkiyenine.

The parameters RMSD, RMSF, RG and the number of intermolecular hydrogen bonds were used to assess the stability of protein–ligand complexes. Based on the above results, Cleomiscosin C in complex with 3CLpro was stable in the whole 100 ns simulation, and had excellent hydrogen bonding, so Cleomiscosin C was more valuable than other compounds.

## Conclusion

In this work, 1047 natural compounds originated from 34 Chinese herbal medicines were carefully studied to develop the potential inhibitors of 3CLpro. Molecular docking results showed that 137 compounds possessed favorable binding affinity toward 3CLpro with docking scores lower than -8.1 kcal/mol. Among these, Cleomiscosin C, (+)-Norchelidonine, Protopine, Turkiyenine, Isochelidonine and Mallotucin A showed good drug-likeness properties by analyzing molecular properties. Besides, Cleomiscosin C and Turkiyenine showed excellent pharmacokinetic profiles *in silico* ADMET analysis. In addition, molecular dynamics simulation results further verified that Cleomiscosin C complex with 3CLpro had higher stability than other compounds. Hence, Cleomiscosin C is considerable hopeful to exploit as a potent 3CLpro inhibitor. However, further *in vitro* and *in vivo* evaluations are needed to validate the computational results for practical application.

**Executive summary**

- A potential 3CLpro inhibitor of SARS-CoV-2 main protease were developed from Chinese herbal.
- Virtual screening was employed to improve the efficiency of lead compound discovery through molecular docking, pharmacokinetic simulation and molecular dynamics simulations.
- Cleomiscosin C shows good binding affinity to 3CLpro, prominent drug-likeness properties, and the Cleomiscosin C and SARS-CoV-2 main protease complex presented high stability, is promising as a potential inhibitor of SARS-CoV-2 main protease.
